# A Comparison of Dental Caries in Urban and Rural Children of the Riyadh Region of Saudi Arabia

**DOI:** 10.3389/fpubh.2019.00195

**Published:** 2019-07-16

**Authors:** Mohammed A. Al-Rafee, Abdullah R. AlShammery, Ali S. AlRumikan, Sharat C. Pani

**Affiliations:** ^1^College of Dentistry, Riyadh Elm University, Riyadh, Saudi Arabia; ^2^Ministry of Health, Riyadh, Saudi Arabia

**Keywords:** dental caries, dental public health, oral health, oral health surveys, Saudi Arabia (KSA)

## Abstract

**Background and Aim:** The data presented in this study aimed to document caries prevalence and severity among a representative sample of 6, 12, and 15 years old Saudi Children.

**Methodology:** The study examined a total of 1,986 school children from 75 schools in urban and rural areas of Riyadh region, Saudi Arabia. Children having one or more dental caries lesion were used to determine prevalence of dental caries. Total number of decayed filled teeth (dft) was used to assess caries severity in the 6 years group while the DMFT was used to assess severity of dental caries at the 12 and 15 years age group.

**Results:** The caries prevalence was 85.77% in the 6 years group, 64.98% in the 12 years group, and 71.35% in the 15 years group. Mean dft was 1.4 (SD ± 1.1). The mean DMFT was 1.72 (SD ± 0.49) at 12 years and 2.66 (SD ± 0.55) at 15 years. Both caries rate and severity were higher in rural areas than urban areas although the differences were not statistically significant.

**Conclusion:** The results of this study suggest that the prevalence and severity of dental caries in Saudi Arabia remain high, when compared to global averages.

## Introduction

The countries of the Arabian Gulf have the highest per capital income in the Eastern Mediterranean region (EMRO) of the World Health Organization (WHO). Despite a high standard of living, the region has had a documented history of poor oral health with studies across the different countries attesting to high caries rates and poor oral hygiene (1).

Saudi Arabia is the largest country in this region and over the past decade there has been a great deal of interest in the evaluation of socioeconomic and cultural indicators of oral diseases and methods to quantify their impact (2, 3). While there have been attempts made to evaluate the oral health status in Saudi Arabia there has not been a unified calibrated examination of oral diseases in the Kingdom since 1993 (4).

Saudi Arabia has a young population with persons under the age of 18 years accounting for nearly 40% of the population. The relationship between dental caries in childhood and caries experience as adults is a well-established one (5, 6). Numerous studies from across the region have confirmed the absence of proper oral hygiene and high caries rates in the samples of children studied (7–11). Furthermore, national statistics show that while over 80% of the Saudi population is concentrated in urban regions, the country is distributed over several rural areas spread across 2,250,000 sq. km; making a comparative assessment of urban and rural centers imperative (12).

The World Health Organization (WHO) recommends that oral health surveys in children be conducted at the ages of 5 years (to assess primary dentition), 12 years (to assess the permanent dentition soon after eruption), and 15 years (to assess the impact of dental caries on the erupted permanent dentition) (13). However, across the Saudi Arabia, children begin school at the age of 6 years making the recording of data in this age group more feasible.

The Oral Health Survey in children aged 6, 12, and 15 years was envisioned as a calibrated data collection exercise that would help clinicians, academics, and policy makers across the region better understand the nature of oral disease. The aim of this study was to determine caries prevalence and severity among children studying in the first grade (~6 years), 6th grade (~12 years), and 9th grade (~15 years) and compare the findings between children studying in urban and rural areas of Riyadh region, Saudi Arabia.

## Methodology

### Ethical Approval

Ethical approval for the study was obtained from the research center of the Riyadh Elm University (then Riyadh Colleges of Dentistry and Pharmacy) and permission to collect data was obtained from the Ministry of Health, Kingdom of Saudi Arabia and Ministry of Education, Kingdom of Saudi Arabia. Informed consent to examine the children was obtained from parents and assent obtained from children prior to examination.

### Allocation of Manpower and Calibration of Examiners

As cultural norms of the Kingdom of Saudi Arabia do not permit the entry of males into female schools and vice-versa, two separate sets examiners (2 males and 2 females) were allocated for calibration. The examiners were calibrated for examination using the protocol laid down by the WHO (14). The inter-examiner kappa scores for primary teeth were 0.884 for decayed teeth (d), 0.891 for filled teeth (f), and 0.901 for the decayed and filled teeth (dft). The kappa scores for permanent teeth were 0.921 for decayed teeth (D), 0.99 for missing teeth (M), and 0.912 for filled teeth with an overall score of 0.941 for the DMFT. The average intra-examiner scores for primary teeth were 0.945 for decayed, 0.881 for filled, and 0.916 for dft.

### Sample Power Calculation and Distribution of the Sample

The required sample based on an assumed true prevalence (based on existing literature) of 0.85, assumed sensitivity and specificity of 0.8 and desired precision of 0.05 and confidence level of 0.95 was 1,843 children. The sample used existing census data from the Riyadh region to extrapolate the desired urban and rural sample (12). In order to account for variations in response rate a sample of 2,000 children 1,500 urban and 500 rural distributed over 81 schools (45 urban and 30 rural) were included in the survey using a stratified cluster sample model. In elementary schools junior grades and senior grades have separate head teachers even though they may often be included in the same physical space. For this reason, a school was defined as an administrative unit, rather than the physical school space. In rural areas there was a lower number of students per school, therefore the number of schools sampled was greater than the proportion of students sampled from each school. Children were identified by using attendance lists provided to the data coordinator (AaR) by the head teachers of each of the selected schools. The school clusters were stratified into three groups; first grade (~6 years), sixth grade (~12 years), and ninth grade (~15 years). An initial visit was followed up by a second visit to ensure that most of the targeted children could be included in the study. Of the targeted 2,000 children we were able to obtain data from 1,986 children giving us an actual power of 0.96.

### Recording of Dental Caries

WHO field examination protocol for examination of teeth was followed (14). Teeth were wiped with a dry piece of gauze before examination and a standardized portable LED with an intensity of. The number of decayed missing and filled permanent (DMF) and the decayed and filled primary (df) teeth were extrapolated from the raw data collected (13). Teeth with secondary caries (filled with decay) were considered as decayed teeth for the purpose of caries surveillance (13). No radiographs were used for the assessment of dental caries.

### Analysis of Dental Caries Data

The prevalence of dental caries was estimated by the number of children with at least one active caries lesion as the numerator and the total number of children as the denominator. Children who had received at least one preventive and/or restorative dental visit were deemed to have had access to dental care. The mean dft and DMFT (for the 6 years group) and DMFT (for the 12 and 15 years age group) were compared between urban and rural children using the Mann Whitney *U*-Test. Caries prevalence was compared between the urban and rural populations using the Kolmogrov–Smirnov *Z*-test. Data was processed using the SPSS ver25 data processing software (IBM SPSS, IBM Corp., Armonk NY, USA).

## Results

### Demographic Data

The sample comprised of 1,986 children (1,440 Urban, 466 Rural) of the 2,000 children whose parents were approached to participate in the study. The schools included 38 primary schools (first grade and sixth grade), 22 high schools (ninth grade), and 15 combined schools (where both junior and senior classes were in the same building). The sample comprised of 33 boys schools and 32 girls schools.

The age of the students in the 6 years age group ranged from 5.9 to 7.1 years (mean age 6.32, SD ± 0.37) with no significant gender differences observed (*t* = 0.789, *p* = 0.897). The age in the 12 years age group ranged from 11. 9 to 13.4 years (mean age 12.45 years, SD ± 0.41) with no significant gender differences observed (*t* = 0.893, *p* = 0.767). In 15 years age group the children were aged between 14.9 and 16.7 years (mean age 15.6 years, SD ± 0.38).

### Caries Prevalence

Overall caries prevalence (percentage of the population with one or more dental caries lesion in a primary or permanent tooth) was 85.77% at 6 years, 64.98% at 12 years (for permanent teeth only), and 71.35% at 15 years (Table 1). There was no significant difference in caries prevalence between urban and rural areas at 6 years (*p* = 0.566), 12 years (*p* = 0.432), or 15 years (0.918). Similarly, no significant differences in caries prevalence were found between males and females at 6 years (*p* = 0.728), 12 years (*p* = 0.654), or 15 years (*p* = 0.300).

**Table 1:** Caries prevalence in the population.

		**Percentage of population with one or more dental caries lesion**			
		**Urban**		**Rural**	
		**Gender**		**Gender**	
		**Male**	**Female**	**Male**	**Female**
Age group	6 years−1st grade	87.21	84.64	86.93	85.21.
	12 years−6th grade (permanent teeth only)	62.51	66.85	63.21	68.22
	15 years−9th grade	70.62	71.52	71.88	71.98

### Dental Caries in Primary Teeth

The decayed filled primary teeth (dft) was recorded only at the 6 years age group in keeping with WHO guidelines. The overall dft at 6 years was 1.4 (SD ± 1.1). Females in rural areas had the lowest recorded dft (0.58). Despite the difference in meant dft, there was a large standard deviation and the Mann-Whitney *U*-test found that the differences were not statistically significant (Table 2). When the urban and rural populations were compared it was observed that there was a lower dft observed in the urban (Mean 1.31, SD ± 1.3) when compared to the rural (Mean 1.521, SD ± 1.1). However, the Mann–Whitney *U*-Test showed that this difference was not statistically significant (*U* = 1577.00, *t* = 0.752).

**Table 2:** Comparison of mean dft between males and females in urban and rural areas.

**Location**	**Gender**	**Mean**	**Std. deviation**	**Mann Whitney *U***	Sig^*^
Urban	Male	1.58	1.16	1427.00	0.058
	Female	0.70	1.32		
	Total	1.31	1.3		
Rural	Male	1.94	1.01	1672.00	0.132
	Female	0.43	0.58		
	Total	1.52	1.1		

* *Calculated using the Mann Whitney U-test*.

### Dental Caries in the Permanent Teeth

Dental caries in the permanent teeth was measured using the DMFT index with mean DMFT of 1.72 (SD ± 0.49) at 12 years and mean DMFT of 2.66 (SD ± 0.55). It was observed that females had lower DMFT scores at both 12 and 15 years. When gender differences were analyzed separately for the urban and rural population it was observed that rural females in the 12 years age group had a higher DMFT than their urban counterparts. However, gender differences were not statistically significant (Table 3). When urban and rural samples were compared it was observed that at both age groups the rural population had a greater DMFT score than the urban population, although these differences were not statistically significant (Table 4). There was a significant increase in the DMFT scores from 12 to 15 years. This was true for both the urban and rural population (Table 5).

**Table 3:** Comparison of DMFT between males and females at 12 and 15 years.

**Location**	**Gender**	**Mean**	**Std. deviation**	**Mann Whitney *U***	Sig^*^
12 years—urban	Male	1.78	0.37	63.00	0.531
	Female	1.54	0.30		
	Total	1.61	0.58		
12 years—rural	Male	1.57	0.78	85.00	0.793
	Female	1.82	0.72		
	Total	1.83	0.41		
12 years—overall	Male	1.69	0.52	212.00	0.670
	Female	1.81	0.46		
	Total	1.72	0.49		
15 years—urban	Male	2.34	0.61	101.00	0.781
	Female	2.55	0.39		
	Total	2.40	0.50		
15 years—rural	Male	2.91	0.67	99.00	0.432
	Female	2.62	0.30		
	Total	2.85	0.28		
15 years—overall	Male	2.70	0.49	153.00	0.516
	Female	2.23	0.45		
	Total	2.66	0.55		

* *Calculated using the Mann Whitney U-test*.

**Table 4:** Comparison of DMFT between urban and rural areas at 12 and 15 years.

**Age group**	**Location**	**Mean**	**Std. deviation**	**Mann Whitney *U***	Sig^*^
12 years−6th grade	Urban	1.61	0.58	484.00	0.412
	Rural	1.83	0.41		
	Total	1.72	0.49		
15 years−9th grade	Urban	2.40	0.50	101.00	0.910
	Rural	2.85	0.28		
	Total	2.66	0.55		

* Calculated using the Mann Whitney U-test.

*Differences are not statistically significant*.

**Table 5:** Comparison of DMFT between the 12 and 15 years age groups.

**Location**	**Gender**	**Mean**	**Std. deviation**	**Mann Whitney *U***	Sig^*^
Urban	12 years	1.61	0.58	22.00	<0.001^**^
	15 years	2.34	0.61		
Rural	12 years	1.83	0.41	17.00	<0.001^**^
	15 years	2.85	0.28		
Overall	12 years	1.72	0.49	64.00	<0.001^**^
	15 years	2.66	0.55		

* Calculated using the Mann Whitney U-test.

** *Differences were significant at p < 0.05*.

## Discussion

Dental caries in Saudi Arabia is being recognized as one of the major public health problems facing the Kingdom (4). Despite the increase in publications on dental caries from Saudi Arabia, the last calibrated region wide study was conducted over two decades ago (15). The Riyadh region is one the largest regions of Saudi Arabia in terms of population. The study aimed to assess dental caries in the methodology prescribed by the WHO, as a precursor to a National Oral Health Survey.

Caries prevalence, or the number of children with at least one dental caries lesion is considered to be a reliable indicator of the extent of a dental caries epidemic. The caries prevalence rate seen in this study was lower than the previous nationwide study done in the 1990s which showed a prevalence of over 90%. Overall the findings of this study show a slightly lower prevalence and severity level than a meta-analysis (4) of caries data but are in keeping with some newer studies in the Kingdom (14, 16, 17). While there has been a massive expansion in both preventive and therapeutic dental services provided across Saudi Arabia over the past decade, the actual utilization of those services and whether they were responsible for the reduction in dental caries are beyond the scope of this study (18). The prevalence and severity of dental caries reported in the current study are in keeping with those of recent observational studies published from Saudi Arabia and UAE (5, 17).

Despite this fact the rates are significantly higher than those reported from Europe and North America (19, 20). The rates reported in the study show that even though there appears to be some reduction in the caries prevalence and severity when compared to previous national surveys, the reduction is not as marked as has been shown in populations in Europe and North America (19–21). This fact should be a source of concern to public health planners in the country. Given the multifactorial nature of dental caries, there are could be several reasons for the low reduction in dental caries including poor oral hygiene, lack of parental awareness and inadequate preventive dental services; trends that have been documented by other studies from Saudi Arabia (11, 12, 22).

One of the recommended protocols for the recording of dental caries in primary teeth is the recording of only decayed and filled primary teeth (dft), omitting the numbers of missing teeth in order to avoid the recording of teeth that might have been lost due to natural exfoliation. An alternative method is to record the decayed, extracted and filled teeth (deft). Our study population with primary teeth was aged between 6 and 7 years, an age when exfoliation of primary teeth begins, which was the reason for the use of dft over deft. One of the limitations of this approach, especially in a high caries population, is that teeth extracted due to dental caries are not recorded. Given this fact it is entirely possible that the dental caries prevalence in the primary teeth is actually higher than the currently reported levels. While this would be in keeping with some earlier studies done in Saudi Arabia (2, 3, 16), it would go against more recent studies from Saudi Arabia that have shown rates of caries prevalence in primary teeth that are in keeping with the current study (9, 10).

The current study used population density (as documented by the Saudi National Census) as an indicator of Urban and Rural areas (15). Although there are several definitions available for Urban and Rural area, this was the measure used in the previous national study in Saudi Arabia and therefore chosen to enable accurate comparison to previous data (3). A significant difference from the previous data has been the reversal in urban and rural dental caries trends. Data from the 1990s had shown that rural Saudi children had significantly lower dental caries rates than their urban counterparts (22). While the current data showed no significant difference, it also showed that in many cases dental caries rates and severity in rural children was greater than their urban counterparts. One explanation for this shifting trend could be the fact that over the past two decades an easy availability of processed foods has meant a change in the eating patterns of rural Saudis (23).

One of the limitations of the study was that it only addressed the prevalence and severity of the disease without looking at sociodemographic variables such as parental education, economic status or family size. Furthermore, the current paper only reports the dental caries status and does not examine the oral hygiene practice or other individual reasons for the high dental caries.

## Conclusion

The results of the study suggest that dental caries prevalence and severity in Saudi Arabia remain high when compared to global averages. Further analysis of the data, especially sociodemographic variables is needed to determine effective caries planning strategies for the Kingdom.

## Data Availability

Additional data will be made available upon reasonable request to the authors.

## Ethics Statement

This study was carried out in accordance with the recommendations of the Institutional Review Board of the Riyadh Elm University, with written informed consent from the parents of all subjects. The parents of all subjects gave written informed consent in accordance with the Declaration of Helsinki. The protocol was approved by the Institutional Review Board of the Riyadh Elm University.

## Author Contributions

MA-R was responsible for the conduct of the study, supervision of examiners, and overall logistical support. ARA was responsible of the conceptualization of the study and calibration of the examiners. ASA was responsible for co-ordination between the schools and the logistical requirements for the study. SP was responsible for the analysis and interpretation of the data. All authors contributed to the preparation of the manuscript.

### Conflict of Interest Statement

The authors declare that the research was conducted in the absence of any commercial or financial relationships that could be construed as a potential conflict of interest.
